# Supplementary material for: Alterations in Hepatocellular Carcinoma-Specific Immune Responses Following Hepatitis C Virus Elimination by Direct-Acting Antivirals
Source: Int J Mol Sci. 2022 Oct 1;23(19):11623. doi: 10.3390/ijms231911623 (PMC9570039; doi:10.3390/ijms231911623)
Supplement: Supplementary file 1 [file ijms-23-11623-s001.zip › ijms-1938502-supplementary.pdf]

Alterations in hepatocellular carcinoma-specific immune responses following hepatitis C virus elimination by direct-acting antivirals

Shihui Li [1], Eishiro Mizukoshi [1](eishirom@m-kanazawa.jp), Kazunori Kawaguchi [1], Miyabi Miura [1], Michiko Nishino [1], Tetsuro Shimakami [1], Kuniaki Arai [1], Taro Yamashita [1], Yoshio Sakai [1], Tatsuya Yamashita [1], Masao Honda [1], and Shuichi Kaneko [1]

[1] Department of Gastroenterology, Kanazawa University，Graduate School of Medicine , Kanazawa, Japan.

Table of contents

Supplementary figures…………………………………………………………………..2

Supplementary Figures

Supplementary Figure S1

Group	No.	Cyp-B$_{109}$	SART2$_{899}$	SART3$_{109}$	p53$_{161}$	MRP3$_{765}$	MRP3$_{692}$	AFP$_{403}$	AFP$_{434}$	AFP$_{357}$	hTERT$_{167}$	hTERT$_{461}$	hTERT$_{324}$	WT-1$_{235}$	EZH2$_{291}$	GPC3$_{298}$	NY-ESO-1$_{158}$	SCCA$_{112}$	IMP-3$_{508}$	Hsp70$_{136}$	CMV pp65$_{328}$
Increased group	1	0/3.5	0/0	0.5/0	1.5/0	0/0	0/0.5	0/0	0.5/0.5	0/0	0.5/3	0/1	0/2	0/0.5	0/5	0/6	0/0	0/0	0/13.5	0.5/3	0/50.5
	2	3/0	0/5	0/0	0/0	1.5/0	0/0	11/16	0.5/4.5	1/0	0/3.5	0/0	0/6.5	2.5/5.5	0/0	0/9.5	0/7.5	1/0	0/105.5	0/8.5	2.5/0
	3	0/5	0/0	0/14.5	0/4.5	0/0	0/0	0/9	0/12.5	0/4.5	0/0.5	0/0	0/10	0/7.5	0/0	0/0	0/0	0/0	0/60.5	0.5/0	9.5/16.5
	4	2/0	1/0	0/0	0/0	1/0	5/0	7/1	4/13	5/59	1/125	2/0	4/2	3/5	8/4	7/0	4/0	1/0	2/20	1/26	0/0
	5	0/1.5	0/2	0/3.5	0/1	0/3	0/3	0/63	0/8	0/3	0/2	0/2	0/2	1.5/4	0/2	0/3	0/2	0/3	0/0	0/38	22.5/83
	6	0/0	0/0	0/0	1/0	0/0	0.5/0	10/76	0/6	0/4	0/5	0/1	0/4	0/7	0.5/8	0.5/1	0/8	0/0	0/5	0/5	0/1
	7	1/0	0/2	4/12	3.5/14	0/6	0/0	0/2	1.5/12	4.5/5	4/7	0/1	1/0	0/11	1.5/22	4/12	0/0	4/0	12/29	4/18	0/6
	8	1.5/4	0/31	0/61.5	0/10.5	0/4	0/0	7.5/8.5	4.5/24.5	0/5.5	0/6	0/7	0/2	0.5/37.5	0/22	0/0	2.5/6	0/7.5	11/14	9.5/23	34/60.5
	9	0/13.5	2/5	8/7	2.5/7.5	6.5/13.5	0/0	2/7.5	0.5/8	5.5/3	2.5/7	8.5/8.5	1/5	7.5/7	3/2	6/10	0/2	0/1	5/29	2.5/14	4/0
	10	4.5/22.5	0/48.5	8/38.5	0/35.5	0/54.5	0/0	1.5/90.5	0/50.5	4/19.5	0/45.5	0/32.5	0/23.5	2/61.5	0/32.5	0/56.5	0/45.5	0/27.5	2.5/129.5	0/6.5	22.5/0
	11	4/0	0/2.5	4/11	1.5/4.5	0/0	6/0	3.5/25	3/9.5	4.5/1.5	1/0	0/0	2/0	19.5/17	4.5/0	1.5/0	1/0	3/0	3.5/0	5/29.5	4.5/0
Mixed group	12	0.5/15	1/4	1/11	3.5/6	21.5/0	2/4	93/46	2.5/8	5/5	1.5/0	22.5/3	3/6	3/16	2.5/8	7/2	23.5/0	6/2	4/27	2/14	31/2
	13	15/0	6/0	19/0	0/0	0.5/0	0.5/0	14.5/1.5	0/0.5	7.5/0	0/1	0/0	0.5/0	3.5/0.5	2.5/0	1.5/1.5	0.5/0	0/0	0/119	0/1.5	10.5/7.5
	14	0/0	5/14.5	0/8.5	0/3.5	2/0	1.5/0	5/6.5	13.5/10.5	9/11.5	0/3.5	0/6.5	5.5/1	17/14	10/2.5	0/9.5	0/13.5	2.5/0	14.5/0	14.5/0	6.5/0
	15	0/0.5	0/0	1/0	0/5.5	0/0	4.5/0	15.5/5	13.5/15.5	3.5/0	4.5/0	0.5/0	1.5/0	12.5/0.5	14.5/3.5	8.5/0	2.5/0	0/0	1.5/0	11.5/54.5	56.5/0
	16	26/2.5	1.5/4	0/3	0/0	0/14	26.5/2	27/105	9/16	0/0	0/1	0/1	0/7	0/12	6/0	1.5/0	34.5/0	0/1	1.5/4	1.5/0	221.5/258
	17	1.5/3	4.5/1.5	6/4.5	3/4	7/4	2.5/2.5	6/25.5	6/3	4.5/2	2.5/0	6/7	0/4	32/3	7/0	8.5/1	0/3	5.5/5	15.5/1	3.5/1	58.5/9
	18	2.5/23	0/10.5	0/6.5	0/1	0/0.5	0/0	0/14	0/23	0.5/6	0/3	0/3.5	0/0	0.5/16.5	0/0	3/3	0.5/2	0/0	147/0	0/31	0/0
	19	2/4	27.5/7.5	45/5.5	1/1	3/0	20.5/0	14.5/4	44.5/9	2.5/13.5	2/2	2.5/0	7.5/0	17.5/4	7.5/0	3/3	1.5/0.5	5.5/0	15/0	10/0	6/1
	20	0/2.5	0/0	0.5/0	0/3.5	0/0	0/0	12/83	2/6.5	0/1	0.5/0	1.5/0	0/0	2.5/4.5	0/0	2.5/0	3.5/0.5	0/3.5	14.5/0	0/0	29.5/20.5
	21	3/11	15/8.5	16/19.5	0/11	0/3.5	7/9	11/11	46/21	2/10	0/11	0/4	0/5	10/14	17/7	6/5	3/5.5	0/5	11/4.5	17/1.5	51/6
	22	10/0	0/2.5	5/0	2.5/5	0/12	0/0	1.5/4.5	6/0	4/0	0/1.5	0/0.5	0.5/0	0/3	0/2	0/2	0/3	2/3	16.5/12	0/3	0/15
	23	16.5/7	455/366.5	4.5/11	0/2.5	0/0	0/0.5	0/4	11.5/9	13/0	3/0	0/0	4.5/0	2.5/3	4.5/0	2/0	8/0	0/0	5/0	6.5/14.5	6.5/0
	24	5.5/6.5	0/6	0/12	0/8	0/2	0/0	2.5/16	2.5/19	0/4	0.5/0	0/0	0/1	4/0	3.5/1	0/0	0/6	0/2	10.5/1	2/2	2/1
	25	0/16	1/18	0/35.5	0/0	0/0	0/0	0/4	3.5/0	0.5/0	0.5/15	1/0	86.5/0	3/6	0/0	4/0	2/0	0/0	1.5/0	3.5/5.5	3/1
	26	0.5/11.5	2.5/2	28.5/4.5	3/1	1.5/4.5	0.5/0	3.5/2.5	6/9	7/3	11/6.5	6.5/8.5	0.5/0	10/5	4/0	7.5/9.5	4/5.5	0.5/7.5	2/0	6.5/89	33/11
	27	3.5/14.5	0/20	2.5/21	0/9	0/19.5	0.5/5	2/16	5.5/26.5	0.5/15	4.5/4	0/5	1.5/5	4/21.5	5/12	0/8	0.5/4	1.5/0.5	15.5/0	6.5/3	4/0
	28	9.5/49	49.5/24.5	42/22	10/20.5	0/11.5	7/9.5	7/19	29.5/30.5	17.5/17	8.5/16	16/7	0/26.5	0/39	0/15	2/8	2/16	0/21	0/0	0/0	0/2
	29	8/10	3/0	6/3	0/0	0/0	0/23	0/1	23/19.5	0/0	0/0	0/0	0/0	0/0	0/0	0/0	0/0	0/0	0/0	102/0	0/0
Decreased group	30	0.5/3.5	0.5/3	0/1	1/0	20.5/1	1/0	2/0	1.5/5.5	3/0	1.5/3	20/3	4/0.5	0.5/2	0/0	0/7	22.5/3	0.5/1.5	0.5/2.5	1.5/6.5	91/3.5
	31	0/6.5	0/5	0/0.5	73.5/3	0/2	0/1.5	0/8.5	45.5/15.5	0/1	3.5/2.5	0/2.5	0/1	2/4	0/3	0/2.5	0/2	0/0.5	0/2.5	0/6.5	0/1.5
	32	2.5/1.5	1/3	3/5.5	2/6	5/4.5	4.5/0	4.5/2.5	0/6	19.5/0	1.5/1.5	3.5/1	0/0	5.5/3.5	2.5/0.5	1.5/2.5	81.5/0	5.5/1.5	0/0	2.5/0	11.5/1.5
	33	0/0	1/1	3.5/3	14/1.5	2/0	0/0	0/4	2/0.5	1.5/0	30/0	1.5/0	0.5/3	1/0	0/0	5.5/0	1.5/0	0/0	0/0	14.5/0	0/0
	34	2/0	112/1	8/0	0/0	0/0	0/0	0/0	0/2.5	2/7	0/0	0/0	0/0	0/5.5	0/0	0/0	0/0	0/0	0/0	1/0	0/0
	35	0/0	0/2	0/2	3.5/0	11/1.5	3.5/0	1.5/1	10/14.5	0/0	12.5/3.5	1.5/4.5	4/4	4/5.5	9/7	0/1	0/1	2/3	61/0	18/0.5	122/113
	36	6.5/3.5	11/2.5	7/4.5	0/1.5	2/0	1.5/1	5/3.5	16/7.5	7/2.5	2/0	1/0	5/0	19/3	0/0	2/0	0/1	0/0	0/0	0/0	26/33
	37	0/0.5	0/5.5	20.5/0.5	8/1	0/1	0/7.5	5/2	17.5/15.5	13/1	0/2	0/3	0/2	0/6	0/1.5	0/1	0/4.5	0/3	0/0	0/3.5	26.5/100
	38	0/0	0/0	0.5/0	1/0	1/0	2.5/0	1.5/0	5/0	2/0	4/0	2/0	1/0	3.5/0	4/0	4/2	5.5/7.5	6.5/0	11.5/0	5.5/0	293.5/291.5
Unchanged group	39	1/1.5	1/2	0/1.5	2/0	0/0.5	0/1.5	0/5	0.5/3.5	0/6.5	0/1	1/0.5	1.5/0	1.5/2	0/4.5	0/4.5	0.5/3.5	0/0	0/0	0/2	101.5/41
	40	1/3	2/2	0.5/0	2.5/0	0.5/0	0/0	8/5	7/7	1/0	3.5/2	0.5/0.5	1.5/2	3.5/5	4/1	2.5/0	0.5/0	0/0	4.5/0	6.5/3	21.5/35
	41	4/0	4.5/0	4.5/0.5	0/0	0.5/0	0.5/0	0/4.5	0/0	1.5/0	0.5/0.5	0/0	0/0	2/0	1.5/0	0/0	0.5/0	2.5/0	0/0	3/1	1.5/0
	42	0/2	0.5/0.5	0/0.5	0/2	1.5/0	3/0	7/2.5	9/0.5	9/3	5/0.5	0.5/2	3/3	2/2	9/0	4/0.5	1/0.5	4.5/0	6.5/0	4.5/0	2.5/0
	43	0/0	3.5/2.5	0/2.5	2/0	1.5/1	0.5/0	0/0.5	1/1.5	0/0	0/0	0/1	0/0	3.5/1	2.5/0	0.5/0	1/0.5	1.5/0.5	2.5/0	0/0.5	0.5/0
	44	0/0	0/0.5	0/0	0/0	0/0	0/0	0/1	0/1.5	0/0	0/0	0/1	0/0	0/7	0/0.5	0/2	0/0	0/0	0/0	0/0	122/0
	45	8/6.5	0.5/0	0/0	0/0	0/0	0/0	2/0	0/0	0/0	0/0	0/0	0/0	0/3.5	0/0.5	0/0	0/0	0/0	0.5/0	2.5/0	0/0
	46	0/1.5	0/0	1/0	0/0	0/0.5	0/0	0/0.5	0/1	0/0	0/1.5	0/0.5	0/1.5	0/0	0/0	0/1	0/1	0/0	0/0	0/0	0/0
	47	0/0	0/0	0/0	0/0	0/0	0/0	0/0	0/8.5	0/0	7/0	0/0	0/0	0/0.5	0/9	0/0	0/0	0/0	0/0	0/0	35.5/4

Figure S1. Immune responses against HCC-related TAA-derived and control peptides in 47 chronic hepatitis C patients before and after the treatment with DAAs

The IFN-γ ELISPOT assay was performed to examine the responses to 19 TAA-derived and CMVpp65 control peptides in 47 patients before and after the treatment with DAAs. Patients were categorized into four groups according to changes in immune responses after the treatment with DAAs, as described in the Materials and Methods: increased group (n=11), mixed group (n=18), decreased group (n=9), and unchanged group (n=9). The number of specific spots before (left side) and after the treatment with DAAs (right side) is shown. Specific spots were calculated by subtracting number of spots in peptide untreated control wells from the number of spots in peptide treated wells. Orange boxes indicate a positive increase, based on the number of

specific spots on the right side being ≥10 and at least two-fold greater than that on the left side. Blue boxes, opposite of orange boxes, indicate a positive decrease, based on the number of specific spots on the left side being at least 2-fold greater than that on the right side with at least 10 spots. Number with red frame: patients with a history of HCC；Number in green color: patients with cirrhosis；Number with yellow background: non-SVR patients.

Supplementary Figure S2

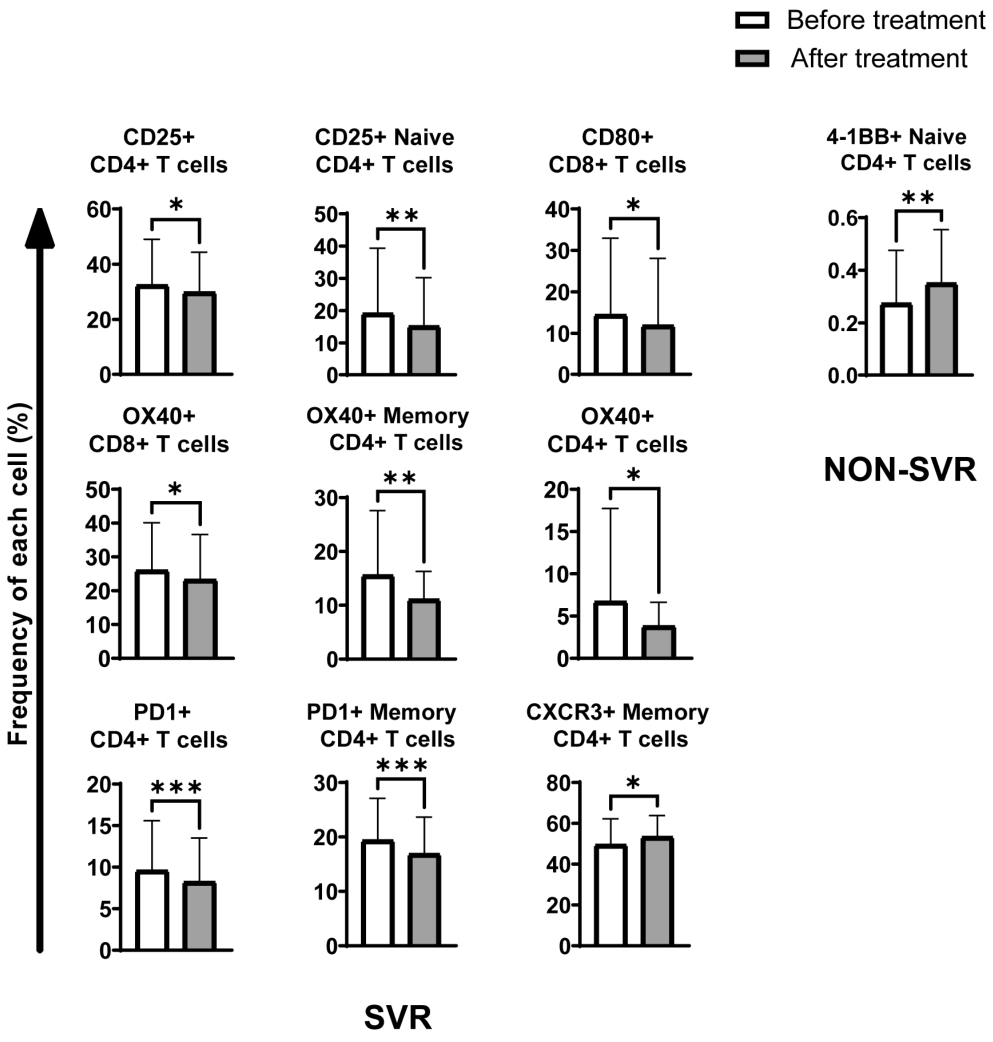

Figure S2. Comparison of the frequency of each T cell subpopulation before and after the treatment with DAAs in SVR and non-SVR groups

This figure shows the rest of the data with significant changes except as noted in Fig. 3. These data are shown as the mean ± SD. The paired *t*-test was used to calculate P values. *P < 0.05, **P < 0.01, ***P < 0.001.

Supplementary Figure S3

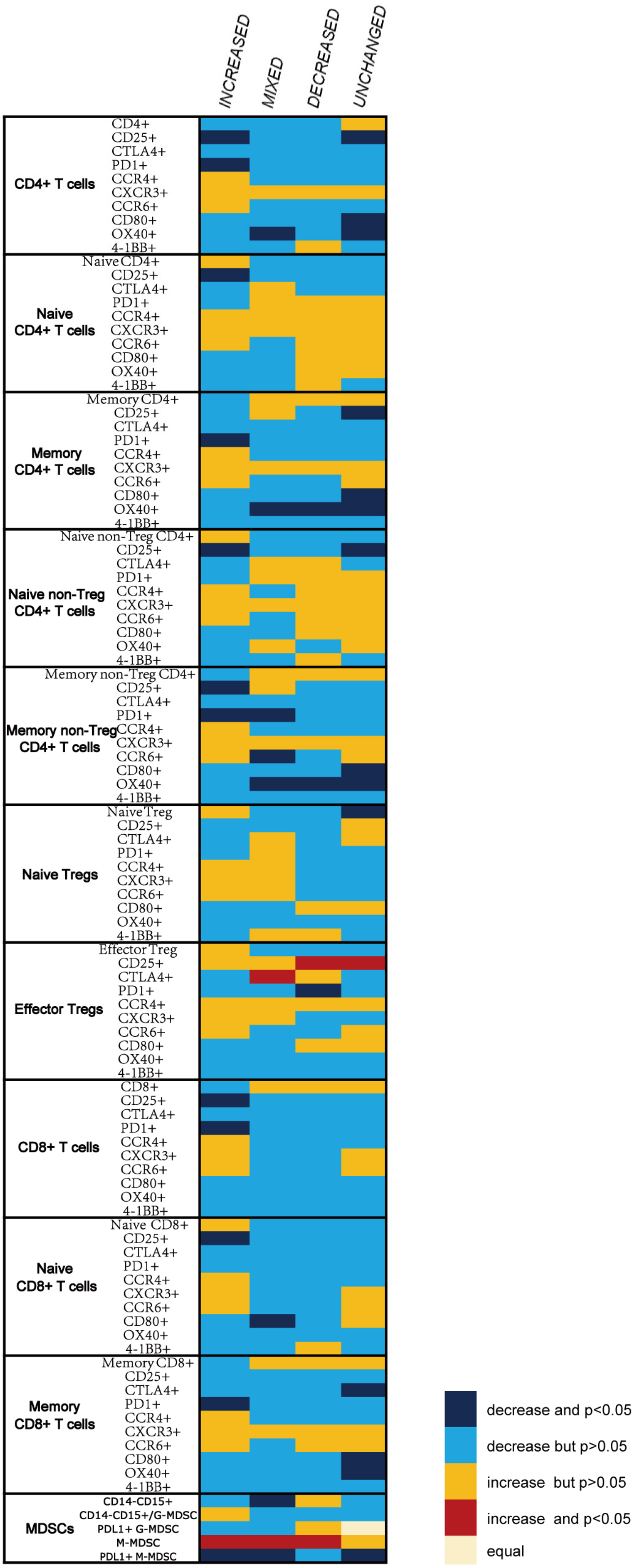

Figure S3. The whole phenotypic variation map before and after the treatment with DAAs in four groups classified based on ELISPOT assay results

In comparisons with the frequency of T cells expressing each molecule before the treatment, changes after the treatment in the four groups are indicated separately by 5 different colors.

Supplementary Figure S4

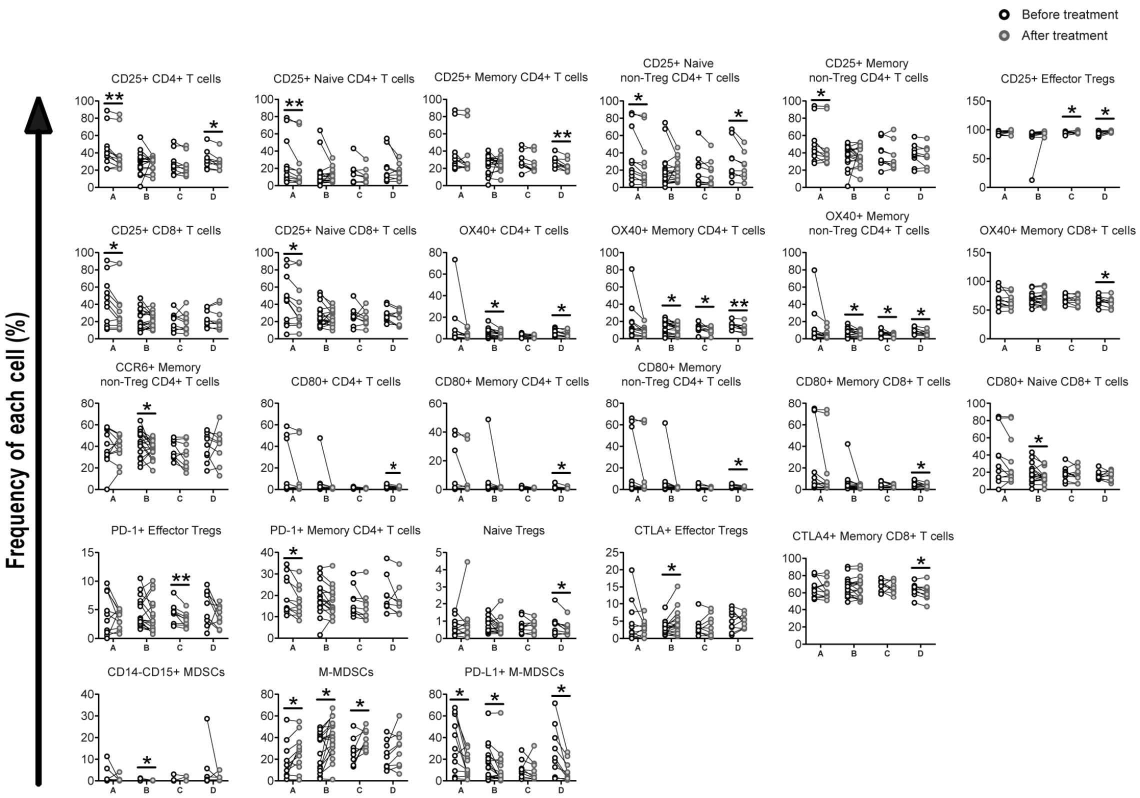

Figure S4. Relationship between TAA-derived peptide-specific immune response changes and the frequency of immune cells with each immune phenotype before and after the treatment with DAAs

Dot plot showing the frequency of each immune cell before and after the treatment with DAA in four groups (A, increased group; B, mixed group; C, decreased group; D, unchanged group). This figure shows the rest of data with significant changes except as noted in Fig. 4B. The paired *t*-test was used to calculate P values. *P < 0.05, **P < 0.01.

Supplementary Figure S5

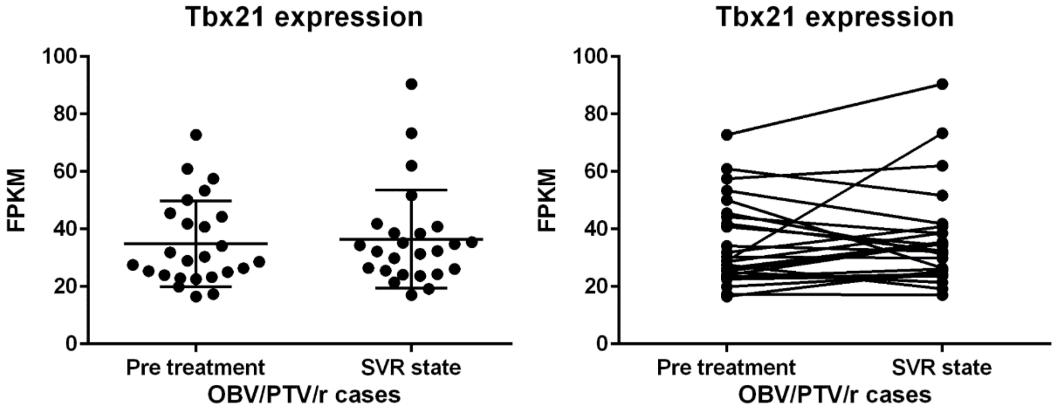

Figure S5. Tbx21 expression levels before and after the treatment with DAAs in chronic hepatitis C patients with sustained virologic responses

RNA was isolated from the PBMCs of 25 patients before and after the OBV/PTV/r treatment. The FPKM values of Tbx21 were calculated from RNA sequence data. Abbreviations: OBV/PTV/r, Ombitasvir / Paritaprevir / Ritonavir; FPKM, fragments per kilobase of exon per million reads mapped.
